# Correction: Attention promotes the neural encoding of prediction errors

**DOI:** 10.1371/journal.pbio.3000368

**Published:** 2019-07-10

**Authors:** Cooper A. Smout, Matthew F. Tang, Marta I. Garrido, Jason B. Mattingley

There are two textual errors within the article. The acronym “MMR” refers to the ‘mismatch response’ ERP component. This acronym has been incorrectly used on page 8 in the PDF. The sentence “followed up this result by averaging MMR amplitudes” should read as follows:

“followed up this result by averaging mismatch response profile amplitudes”.

The acronym and subscript *B*_01_ should be changed to *BF*_10_ on page 14 of the PDF. The following sentence has been revised to include the correct acronym and subscript, *BF*_10:_

“Bayes factors allow for quantification of evidence in favour of either the null or alternative hypothesis, with *BF*_10_> 3 indicating substantial support for the alternative hypothesis and *BF*_10_ < 0.33 indicating substantial support for the null hypothesis”

There is one textual error in the legend of S1 Fig (page 19 of the PDF). The sentence “(G) Genuine MMR during the dot task (ignored deviants minus ignored standards)” should read as follows:

“(I) Genuine MMR during the dot task (ignored deviants minus ignored standards)”

There is an error in the author affiliations. Jason B. Mattingley’s affiliations should list the Australian Research Council Centre of Excellence for Integrative Brain Function instead of School of Mathematics and Physics, University of Queensland. The author byline should read:

Cooper A. Smout^1,2^*, Matthew F. Tang^1,2^, Marta I. Garrido^1,2,3,4^, Jason B. Mattingley^1,2,5,6^

1 Queensland Brain Institute, University of Queensland, Brisbane, Australia,

2 Australian Research Council Centre of Excellence for Integrative Brain Function, Victoria, Australia,

3 School of Mathematics and Physics, University of Queensland, Brisbane, Australia,

4 Centre for Advanced Imaging, University of Queensland, Brisbane, Australia,

5 School of Psychology, University of Queensland, Brisbane, Australia,

6 Canadian Institute for Advanced Research (CIFAR), Toronto, Ontario, Canada

*cooper.smout@gmail.com
