# Supplementary figures and images for: Attention promotes the neural encoding of prediction errors
Source: PLoS Biol. 2019 Feb 27;17(2):e2006812. doi: 10.1371/journal.pbio.2006812 (PMC6411367; doi:10.1371/journal.pbio.2006812)

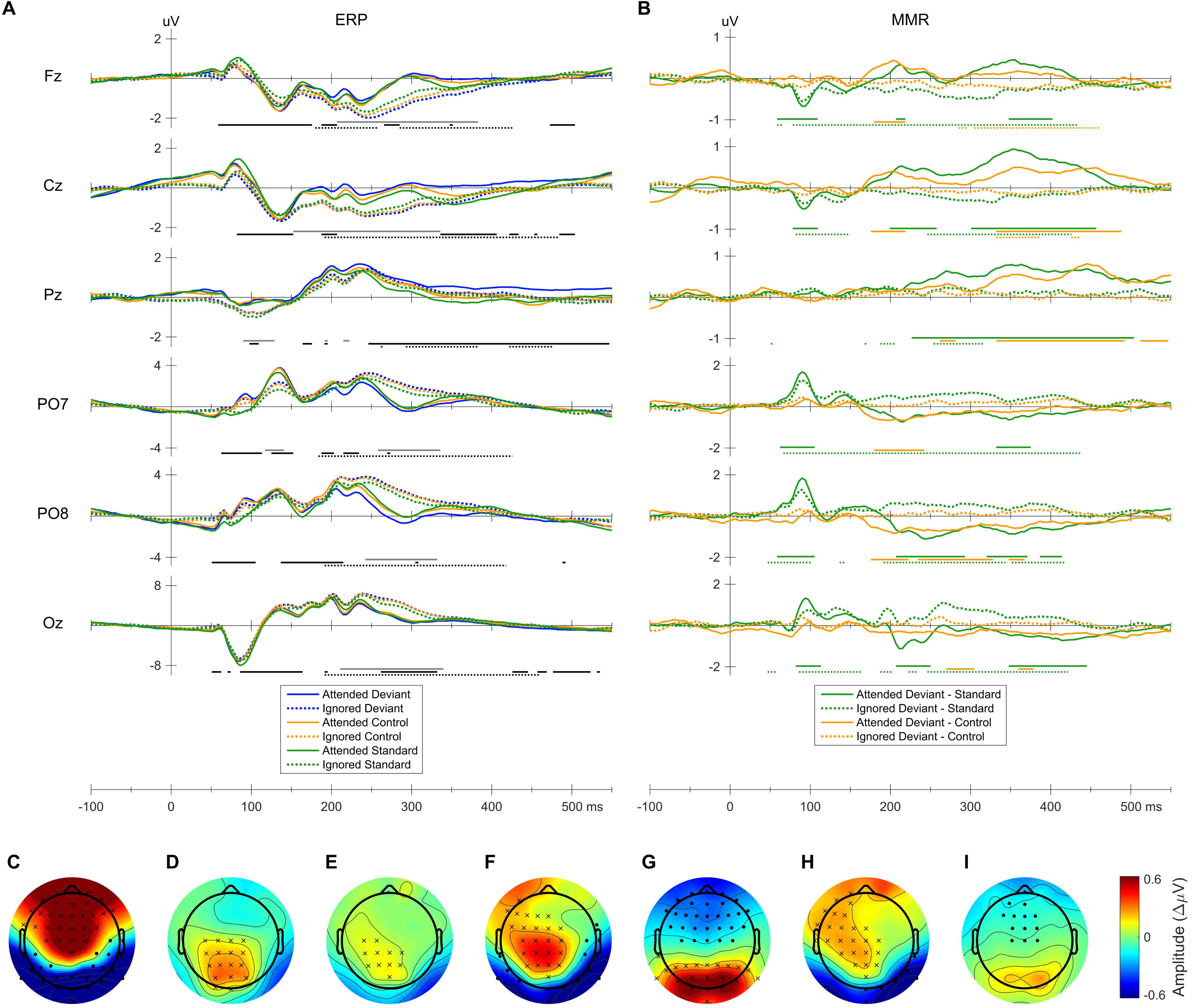

Supplement: S1 Fig — (A) ERPs at selected electrodes, shown separately for each condition. Bars underneath each plot indicate time points at which there was a significant main effect of attention (solid grey bar), significant main effect of prediction (solid black bar), or a significant interaction between attention and prediction (dotted black bar) at the plotted electrode. (B) Classic MMR (deviants minus standards) and genuine MMR (deviants minus controls) at selected electrodes, plotted separately for each level of attention. Green and yellow lines denote the classic MMR and genuine MMR, respectively; solid and dashed lines denote attended and ignored stimuli, respectively. Bars underneath each plot indicate timepoints at which there was a significant MMR in the corresponding condition, at the plotted electrode. Attended deviants were significantly different from attended standards (39–504 ms, cluster-corrected p < 0.001) and attended controls (172–550 ms, cluster-corrected p < 0.001). Ignored deviants were significantly different from ignored standards (47–438 ms, cluster-corrected p < 0.001) and ignored controls (285–461 ms, cluster-corrected p = 0.001) (C–I) Topographies of effects collapsed across time points between 200 and 300 ms. Asterisks and dots denote electrodes with larger or smaller responses, respectively, in at least 25% of the displayed time points. (C) Main effect of attention (attended minus ignored). (D) Classic MMR (deviants minus standards). (E) Genuine MMR (deviants minus controls). (F) Classic MMR during the grating task (attended deviants minus attended standards). (G) Classic MMR during the dot task (ignored deviants minus ignored standards). (H) Genuine MMR during the grating task (attended deviants minus attended controls). (G) Genuine MMR during the dot task (ignored deviants minus ignored standards). ERP, event-related potential; MMR, mismatch response. (TIF) [file pbio.2006812.s001.tif]

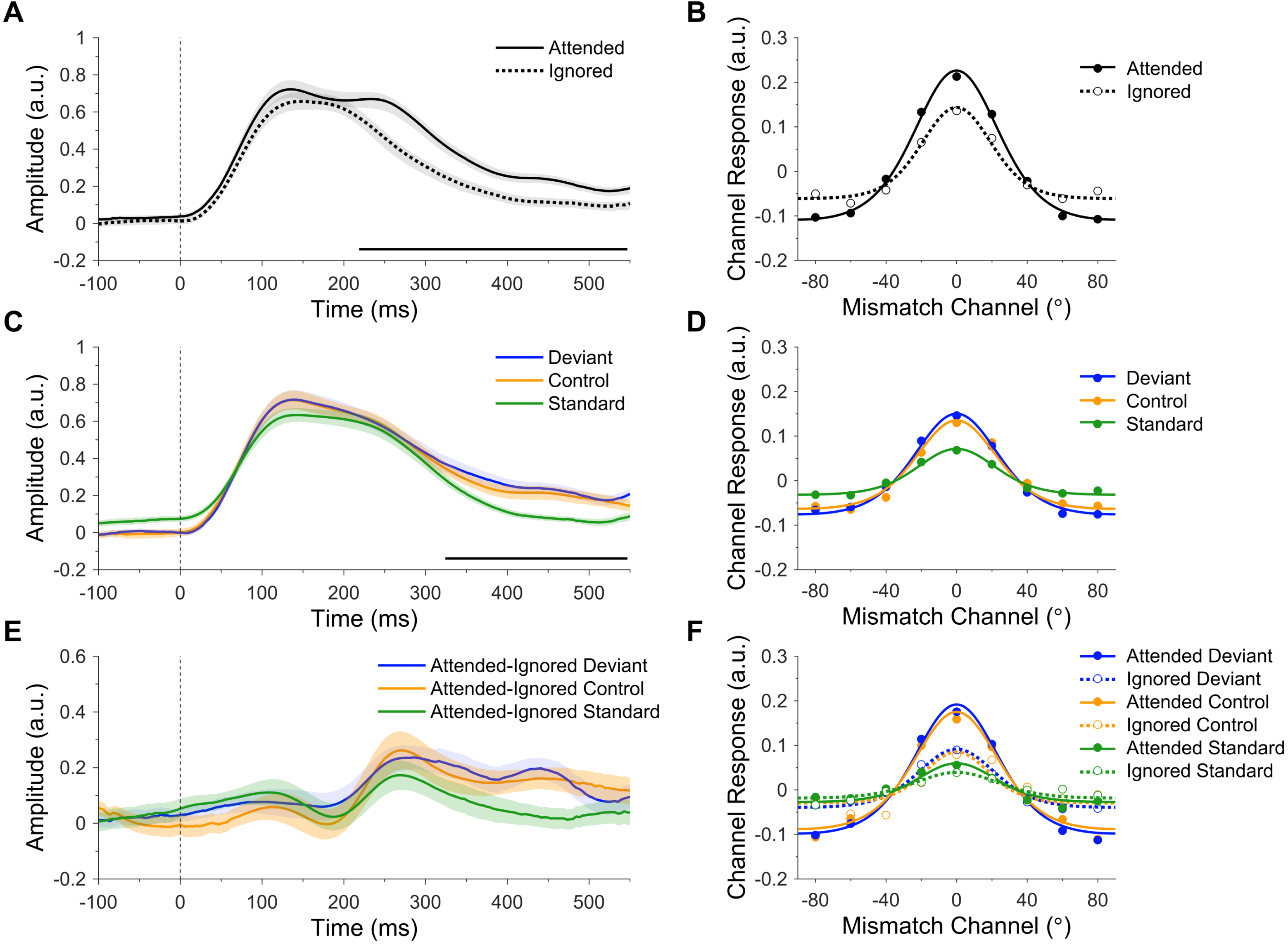

Supplement: S2 Fig — (A) Main effect of attention on orientation response profiles. The amplitude of attended gratings was larger than that of ignored gratings (219–550 ms, cluster-corrected p = 0.001). Shading denotes standard error of the mean. The black bar along the x-axis denotes significant time points. (B) Orientation response profiles, collapsed across significant time points in A. Dots show activation in each of the nine modelled orientation channels. Curved lines show the functions used to quantify the amplitude and concentration of orientation-tuned responses (fitted to grand average data for illustrative purposes). (C) Main effect of prediction on orientation response profiles (black bar along the x-axis denotes significant time points, 324–550 ms, cluster-corrected p < 0.001). The amplitude of standards was reduced relative to both deviants and controls. (D) Orientation response profiles, collapsed across significant time points in C. (E) Interaction between attention and prediction on orientation response profile amplitude. Time-courses show the effect of attention (attended minus ignored) on each stimulus type. (F) Orientation response profiles, collapsed across time points in the nonsignificant but trending cluster in E (414–481 ms, not displayed, cluster-corrected p = 0.093). (TIF) [file pbio.2006812.s002.tif]

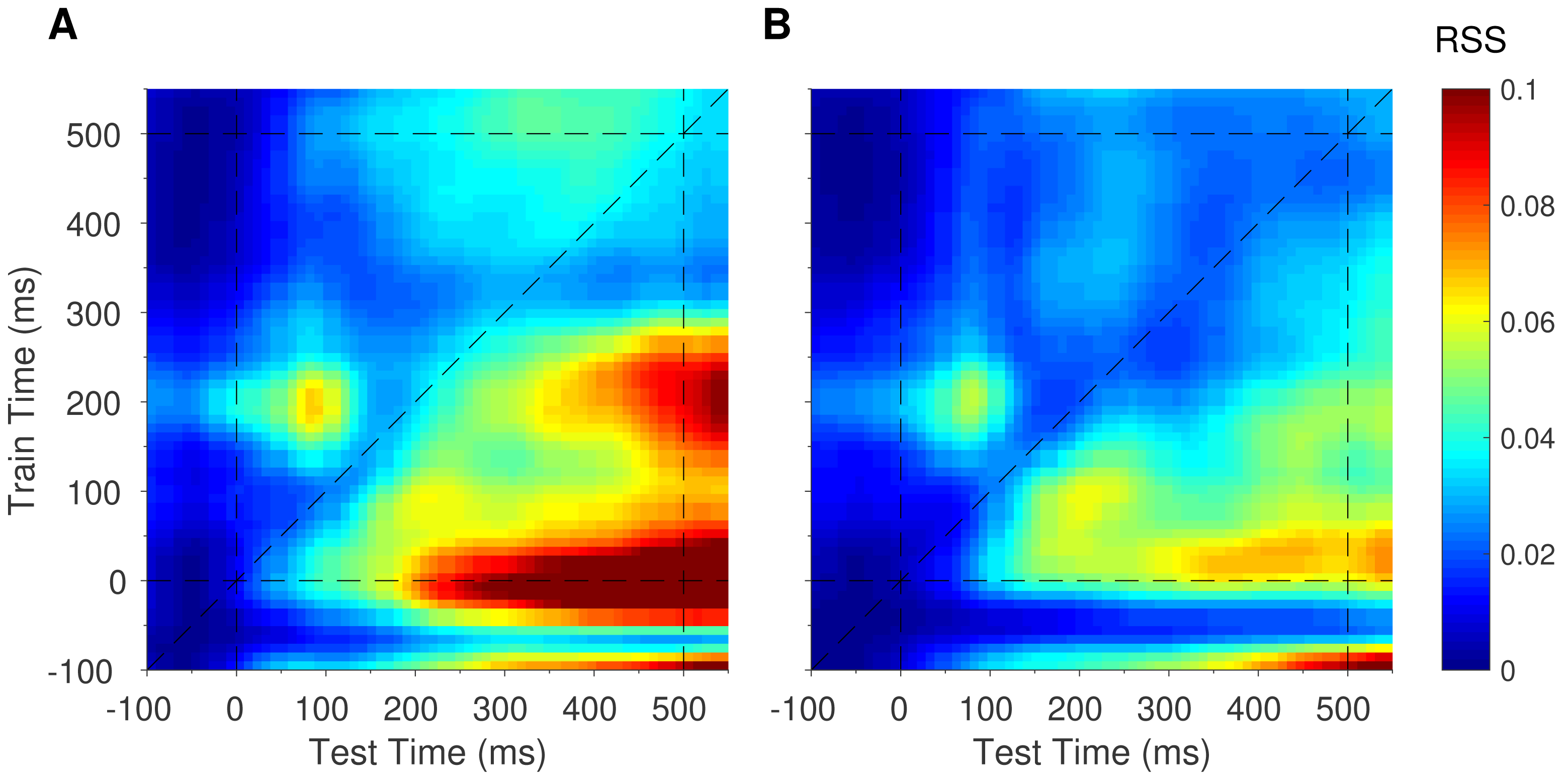

Supplement: S3 Fig — Note the high RSS values along the x-axis beginning at 200 ms, indicating that the apparent generalisation of spatial maps trained at stimulus onset to later times in the epoch (Fig 5, red patch along the x-axis) was likely due to noise. RSS, residual sum of squares. (TIF) [file pbio.2006812.s003.tif]
